# Assessment of circulating copy number variant detection for cancer screening

**DOI:** 10.1371/journal.pone.0180647

**Published:** 2017-07-07

**Authors:** Bhuvan Molparia, Eshaan Nichani, Ali Torkamani

**Affiliations:** 1 The Scripps Translational Science Institute, La Jolla, CA, United States of America; 2 The Department of Integrative Structural and Computational Biology, The Scripps Research Institute, La Jolla, CA, United States of America; 3 Massachusetts Institute of Technology, Cambridge, MA, United States of America; 4 The Department of Molecular and Experimental Medicine, The Scripps Research Institute, La Jolla, CA, United States of America; 5 Scripps Health, La Jolla, CA, United States of America; CNR, ITALY

## Abstract

Current high-sensitivity cancer screening methods, largely utilizing correlative biomarkers, suffer from false positive rates that lead to unnecessary medical procedures and debatable public health benefit overall. Detection of circulating tumor DNA (ctDNA), a causal biomarker, has the potential to revolutionize cancer screening. Thus far, the majority of ctDNA studies have focused on detection of tumor-specific point mutations after cancer diagnosis for the purpose of post-treatment surveillance. However, ctDNA point mutation detection methods developed to date likely lack either the scope or analytical sensitivity necessary to be useful for cancer screening, due to the low (<1%) ctDNA fraction derived from early stage tumors. On the other hand, tumor-derived copy number variant (CNV) detection is hypothetically a superior means of ctDNA-based cancer screening for many tumor types, given that, relative to point mutations, each individual tumor CNV contributes a much larger number of ctDNA fragments to the overall pool of circulating free DNA (cfDNA). A small number of studies have demonstrated the potential of ctDNA CNV-based screening in select cancer types. Here we perform an in silico assessment of the potential for ctDNA CNV-based cancer screening across many common cancers, and suggest ctDNA CNV detection shows promise as a broad cancer screening methodology.

## Introduction

According to the National Cancer Institute, 5-year survival rates of cancer patients are the highest when cancer is detected and treated at an early, localized, stage. Currently, there are a number of different cancer-type specific biomarkers used to indirectly detect cancer at an early stage; however most of them are associated with alarmingly high false positive rates (FPRs). For example, ovarian cancer screening using the CA-125 biomarker [1] along with transvaginal ultrasonography has a sensitivity of ~90% but a FPR of 57%[2]. Mammography for breast cancer screening has a FPR of 40–60% over 10 years of screening [3], Cologuard^**®**^ for colorectal cancer screen has a FPR of 13.4% [4], and PSA for prostate cancer screening has a FPR of 20–30% when the test aims to detect >80% of cancers [5]. False positive results, and sometimes screening methods themselves, tend to lead to invasive and uncomfortable procedures that are associated with risk to otherwise healthy individuals; e.g. radiation exposure during mammography and surgery or biopsy in the case of other tumor types. These unnecessary procedures, unfortunately, lead to adverse events in approximately 15% of cases [6]. High false positive rates along with high adverse event rates for follow-up procedures place a significant proportion of the healthy population at unnecessary risk. Thus, an alternative and highly accurate non-invasive method for early cancer detection would both reduce the rate and impact of false positive results on otherwise healthy individuals, and could lead to substantial improvements in survival and quality of life of cancer patients. One possible approach is to utilize circulating tumor DNA (ctDNA) as a causal, rather than correlative, biomarker for the detection of cancer.

A series of studies have demonstrated the ability to detect tumor derived genetic aberrations in circulating free DNA (cfDNA) [7–10]. Point mutation detection methodologies are either unbiased but have modest sensitivity or are high sensitivity but must be customized to a cancer patient based on their known tumor mutation profile–which is not possible for screening purposes. Nevertheless, these methods are extremely useful for cancer treatment monitoring. The major obstacle to the extension of cfDNA tests to cancer screening is the low fraction of ctDNA within cfDNA at early stages. The fraction of ctDNA in the bloodstream varies depending on the cancer burden and the type of cancer but is usually <10% even at late stages, and is generally below 1% ctDNA fraction at early stages when cancer screening offers the most clinical benefit [8, 11–13]. For example, at 0.1% ctDNA fraction of total cfDNA, one can expect only 1–5 ctDNA copies per point mutation per mL of blood [8]–a mutation fraction that is below the error rate of modern next generation sequencing (NGS).

Copy number variations (CNVs), like point mutations, are common and causal for a large proportion of cancer types [14, 15]. Unlike SNVs, tumor derived CNVs contribute to the total cfDNA a much larger number of ctDNA fragments per CNV event (proportional to the size of the CNV), and their detection is not bounded by the error rate of available sequencing instruments but rather the throughput and biases of those instruments. Thus, detection of large circulating tumor-derived CNVs via cfDNA sequencing is potentially a more viable approach to cancer screening. Moreover, recent studies have found that CNV signatures are more likely to be useful for determining the tissue of origin of a tumor, a characteristic that is important for clinical follow-up in a broad cancer screening setting [16–18], primarily due to the fact that somatic point mutations occur recurrently across disparate tumor types.

A few proof-of-concept studies have explored the possibility of tumor derived CNV detection in cfDNA, but these studies have focused on detection of CNVs from a known cancer type and/or were performed on subjects with relatively high tumor loads [12, 13, 19, 20]. A broad assessment of the utility of tumor derived CNV detection in cfDNA as a cancer screening tool has not yet been performed. In this light, we explore the potential for ctDNA CNV detection for cancer screening by evaluating the ability to detect cancer and identify cancer type via large tumor CNV events. We demonstrate that, for many tumor types, including those not necessarily enriched with CNV events, it is theoretically possible to both accurately detect and classify tumor types via large ctDNA detectable tumor-derived CNVs.

## Results

### Theoretical power for detecting ctDNA CNVs

Relatively recent findings of cancer in asymptomatic pregnant women undergoing non-invasive prenatal testing (NIPT) highlight the potential of cfDNA sequencing for cancer screening [21, 22]. NIPT tests are generally powered to detect large chromosomal aberrations, though it has been shown that smaller size CNV events, ~7 Mb in size, can be detected via NIPT testing with greater than 95% sensitivity and specificity [23]. However, NIPT tests utilize low genomic coverage (<1X) and are performed in a context where on average ~10% [24, 25] of the cfDNA in a pregnant woman’s bloodstream is derived from the fetus. In contrast, ctDNA fractions can be 1% or lower for early stage tumors [9]. Moreover, pathogenic cancer CNVs can range anywhere from 1 Mb, to 5 Mb, to 100+ Mb. Therefore, we first determined the theoretical sequencing depth requirements necessary to detect cancer derived CNV events in cfDNA at relevant ctDNA fractions and cancer CNV event sizes.

**Fig 1:**
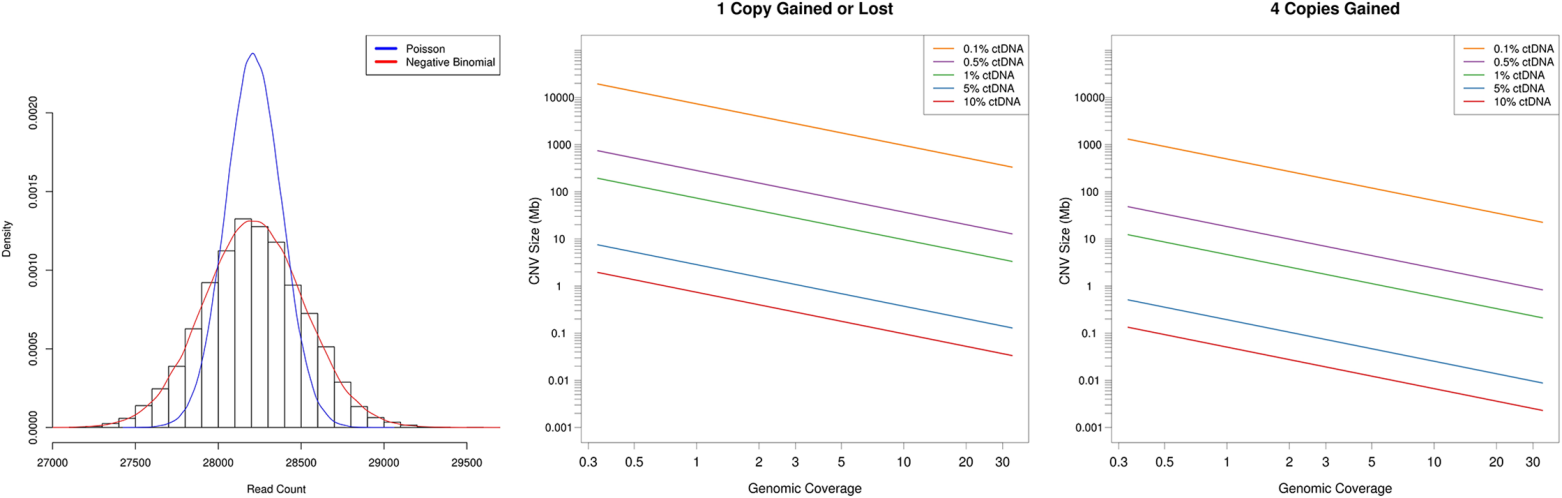
Theoretical limits of ctDNA CNV detection. A) Density plot for healthy donor cfDNA sequencing reads mapped to 10kb genomic bins. A negative binomial (red) and Poisson (blue) distribution was fit to the resultant data. B) The ctDNA CNV size limit of detection (in megabases) is plotted as a function of sequencing depth for single copy change at various ctDNA fractions. C) Same as panel B but for four copies gained.

To determine the relevant statistical model for ctDNA CNV detection, we collected and sequenced cfDNA from 9 healthy donors. Sequence data was mapped to 10kb genomic bins (S1 Fig), outlier bins removed (S2 Fig), bin counts corrected for GC content (S3 Fig) (see *Methods*) and the distribution of bin counts fit to a negative binomial and Poisson distribution (Fig 1A). Visual inspection and goodness of fit analysis determined that the negative binomial distribution was the appropriate statistic for ctDNA CNV detection (Χ-squared, p-value = 0.40).

Next, we determined the theoretical sequencing depth required for ctDNA CNV detection. The relationship between CNV size, ctDNA fraction, and read depth was explored via the negative binomial statistical model. Variance and size parameters of the model were empirically determined from healthy donor cfDNA sequencing as mentioned above (see *Methods*). As can been seen in Fig 1B, assuming first that somatic CNVs are only a single copy gained or lost, at 10% ctDNA fraction, focal gene amplifications, i.e. CNVs as small as 1Mb, can be easily detected with just a 1X coverage of the genome. At 1% ctDNA fraction, large focal CNVs [26], i.e. CNVs of ~30Mb, are detectable at 3X genomic coverage. At 0.5% ctDNA fraction, chromosome arm level changes, i.e. CNVs of ~100Mb, are detectable at 3X genomic coverage. And at 0.1% ctDNA fraction, approximately 131X genomic coverage is required to detect 100Mb single copy changes.

Cancer CNVs, especially amplifications, often exceed a single copy gained or lost [27]. The sensitivity of ctDNA CNV detection increases substantially for highly amplified (≥4 copies gained) regions (Fig 1C). For example, at 0.5% ctDNA fraction and 3X genomic coverage, the detection limit improves from ~100Mb when a single copy is gained to ~8Mb for gains of 4 copies. Similarly, ~100 Mb events become detectable at 10X genomic coverage at 0.1% ctDNA fraction. Thus, the necessary depth to detect cancer CNVs in ctDNA can vary widely from 1X in the best case scenario (high ctDNA fraction + high copy number) where small gene level events are detectable, to >100X in the worst case scenario (low ctDNA fraction and single copy number changes), when only chromosome arm level events (~100 Mb) are detectable.

### Simulation of ctDNA CNV data

Given that the limit of detection for ctDNA CNVs depends heavily on a variety of parameters, we evaluated the potential of ctDNA CNV based cancer screening under the assumption of best case (sensitivity to detect >5 Mb events) and worst case (sensitivity to detect only >100 Mb events) ctDNA CNV resolution scenarios. ctDNA detectable CNV profiles were simulated from The Cancer Genome Atlas (TCGA) data. The human genome was divided into 5 and 100 Mb segments, and for each sample, the average copy number was determined for each segment. For example, if the primary data for a 5 Mb segment of a particular tumor sample included just a 1 Mb subsection with 5 extra copies gained (i.e. 1 extra copy on average), that 5 Mb segment would be labeled as detectable in the best case scenario. Similarly, if the entirety of the 5 Mb bin was spanned by a CNV event of 1 copy number gain, it would also be labeled as detectable in the best case scenario. By the same token, in the worst case scenario, the average copy number of an entire 100 Mb segment must be equal to or exceed 3 copies (average of one copy gained throughout the entire 100 Mb segment) for it to be deemed a detectable event. While more complex event detection and change point detection methods are possible, this approach should represent a lower bound for performance under the given assumptions for ctDNA CNV event detectability.

**Fig 2:**
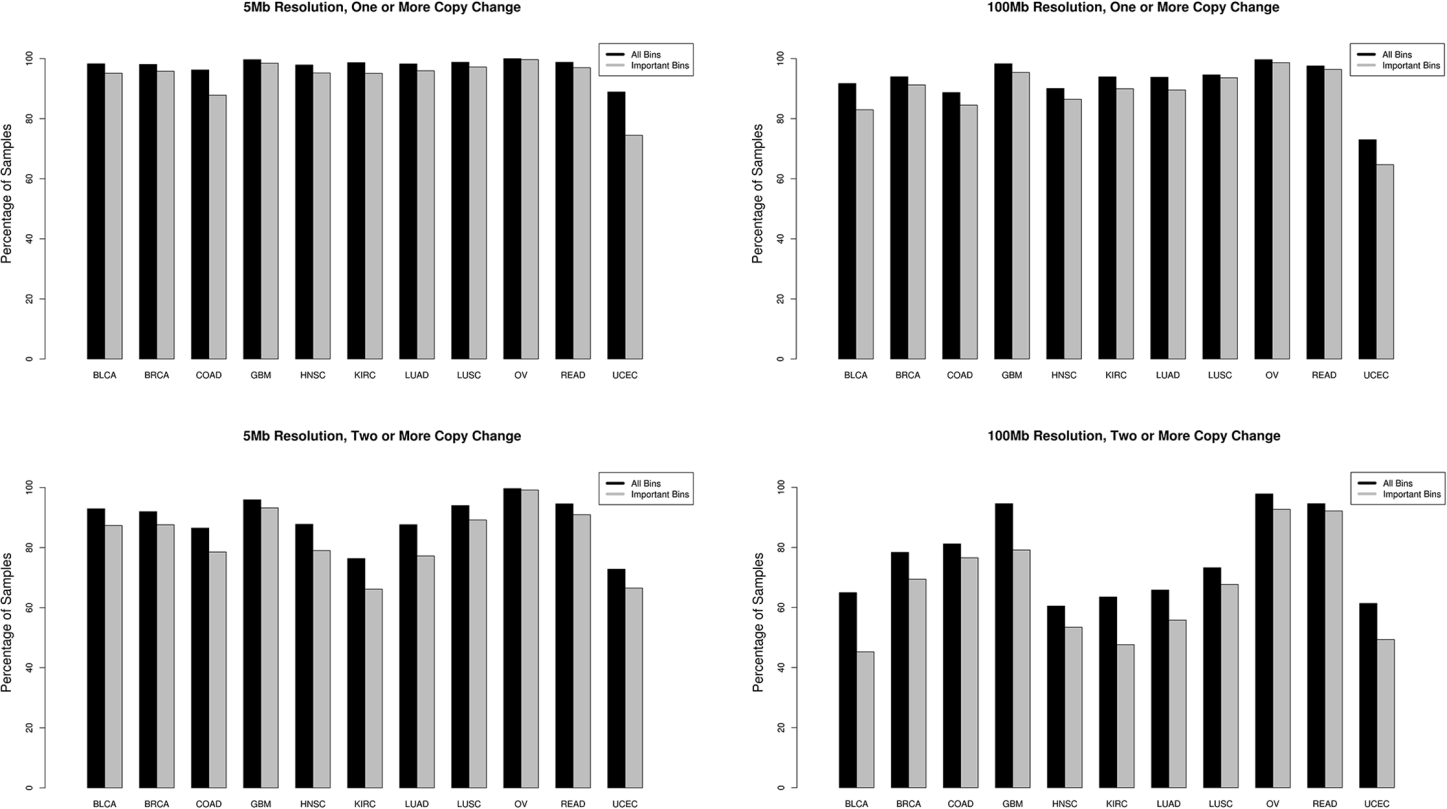
Percentage of cancer samples with ctDNA detectable CNV events. The fraction of samples with at least one detectable CNV event (top panels) or two and more detectable CNV events (bottom panels) at 5 Mb (left panels) and 100 Mb (right panels) resolution are plotted per cancer type. All CNV events were considered (black) as well as only those deemed important for cancer type discrimination by our random forest model (grey).

The simulation was performed for 25 different cancer types (see Methods) but the results we present here are for 11 major pan-cancer [17] solid tumor types: breast adenocarcinoma (BRCA), lung adenocarcinoma (LUAD), lung squamous cell carcinoma (LUSC), uterine corpus endometrial carcinoma (UCEC), glioblastoma multiforme (GBM), head and neck squamous cell carcinoma (HNSC), colon and rectal carcinoma (COAD, READ), bladder urothelial carcinoma (BLCA), kidney renal clear cell carcinoma (KIRC), and ovarian serous carcinoma (OV), for simplicity of visualization; full results are presented in supplemental materials. The fraction of samples with at least one detectable event in the best (Fig 2A and 2B) and worst (Fig 2C and 2D) case resolution scenarios is plotted in Fig 2.

### Classification of cancers based on ctDNA CNV profiles

We utilized the simulated ctDNA CNV profiles to answer two questions: 1) Can ctDNA CNV profiles be used to detect the presence of cancer in general (i.e. differentiate cancer samples from normal samples)? 2) If cancer is detected, can the ctDNA CNV profile be used to determine the tissue of origin for follow-up screening? In order to avoid over-stating the performance of this approach to cancer screening, we present results under best and worst case sensitivity scenarios utilizing both simple and sophisticated classification methods.

**Fig 3:**
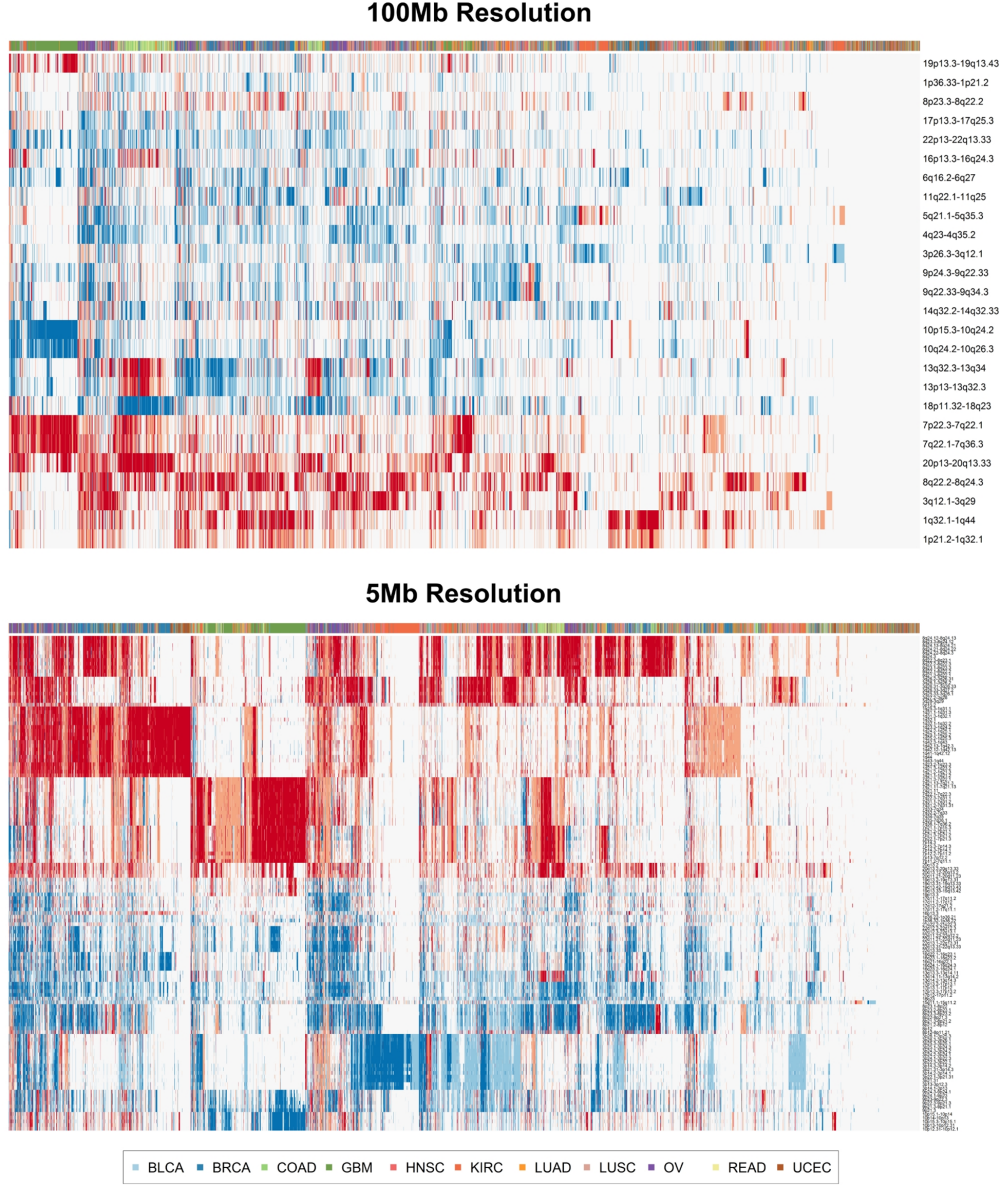
Unsupervised cancer sample clustering with ctDNA detectable CNV events. Heat maps representing the results of unsupervised clustering of cancer samples using 100 Mb resolution (top panel) and 5 Mb resolution (bottom panel) of ctDNA CNV events. Deletions are depicted in blue and amplifications are depicted in red.

First, we explored whether simple clustering of cancer samples based on ctDNA detectable CNV events would be sufficient to discriminate cancer types from one another. Unsupervised clustering utilizing an unfiltered set of all detectable genomic segments did not effectively separate tumor types from one another (results not shown). Therefore, we performed clustering utilizing only genomic segments deemed relevant for distinguishing tumor types from one another in the random forest classification model described below. The resulting heat maps demonstrate some degree of separation of disparate tumor types from one another in both the 5 Mb and 100 Mb ctDNA CNV resolution scenarios (Fig 3). Certain tumor types like GBM and KIRC form cohesive, though not complete, blocks of clustered samples, while most others demonstrated a tendency to form close but intermixed clusters with other tumor types. These results suggest that classification of tumor type by ctDNA CNV profile is feasible, but would require more sophisticated methodology to account for CNV profile heterogeneity within tumor types and similarity across tumor types [17].

**Fig 4:**
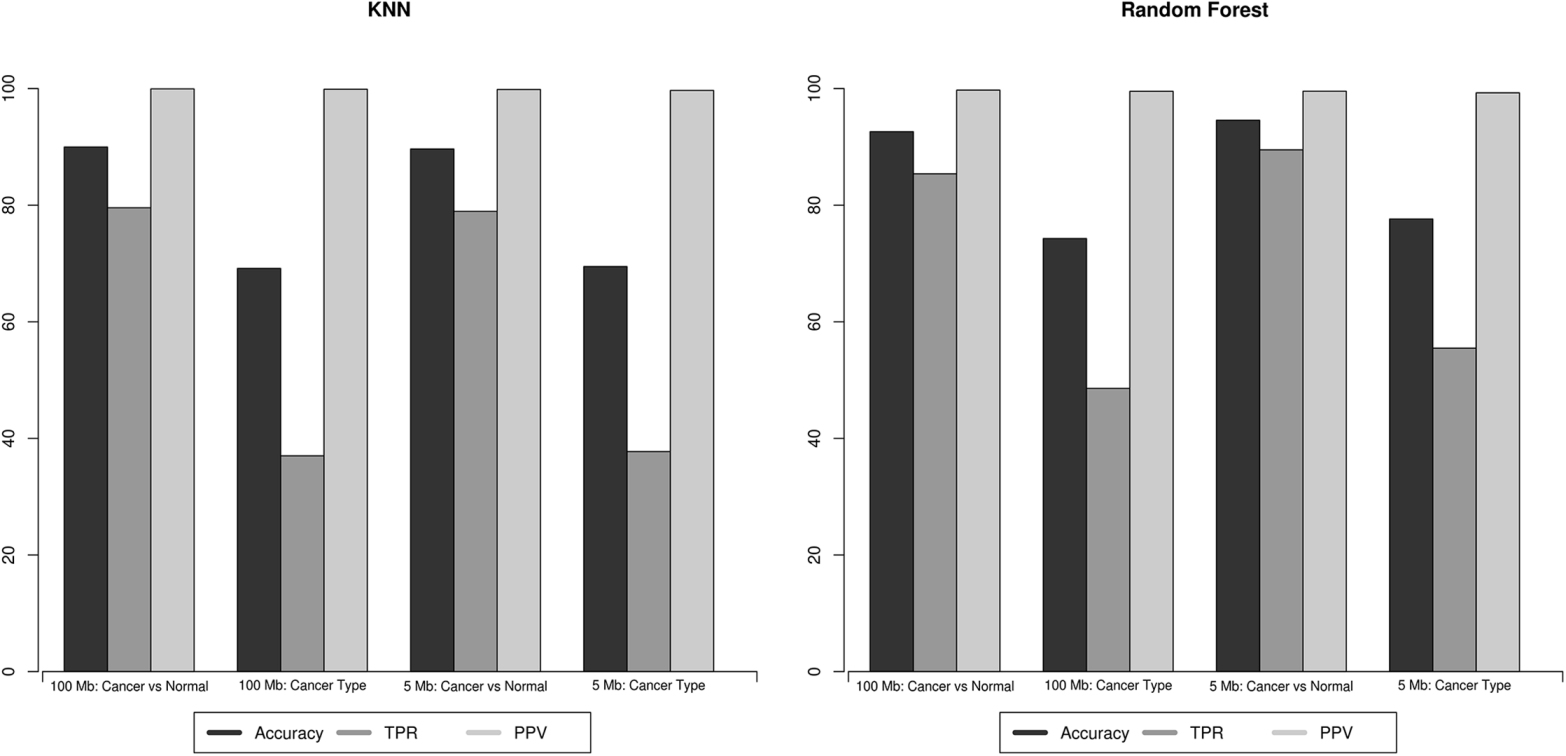
Performance of the classification models. The predictive performance of the KNN model (left panel) and the random forest model (right panel) is plotted. In general, random forest models outperform the KNN models. Positive predictive value (light gray) (PPV) remains stable across models and CNV size resolution. Accuracy (black) and true positive rate (dark gray) (TPR) remain stable at 5Mb and 100Mb resolutions for the KNN model but increase at 5Mb resolution for the random forest model.

Therefore, we evaluated the performance of a simple k nearest neighbor classification approach (see *Methods*). This model was readily capable of identifying the presence of cancer with a true positive rate (TPR) of 0.80 and a positive predictive value (PPV) of 0.999 at the 100 Mb ctDNA CNV resolution; suggesting that, in theory, even a low resolution ctDNA CNV profile can be utilized to effectively detect the presence of cancer with a negligible false positive rate (Fig 4A). At 5 Mb ctDNA CNV resolution, the performance remains unchanged (TPR 0.79, PPV 0.999), suggesting that low resolution ctDNA CNV profiling is sufficient if the goal is to simply detect the presence of cancer (S1 Table).

When the nearest neighbor approach was utilized to determine cancer type, an overall accuracy of 0.69 was observed at 100 Mb resolution (Fig 4A). Again, the overall accuracy did not improve with improved ctDNA CNV resolution (0.69 accuracy at 5 Mb resolution). Certain CNV poor cancer types, such as pancreatic adenocarcinomas, prostate adenocarcinomas and thyroid carcinomas formed a floor for accuracy that could not be improved with increased ctDNA CNV detection resolution (Table 1 and S1 Table).

**Table 1:** Performance of the KNN and random forest classification models for determination of cancer type.

	K-Nearest Neighbor				Random Forest			
	100 Mb		5 Mb		100 Mb		5 Mb	
Sample Type	TPR	PPV	TPR	PPV	TPR	PPV	TPR	PPV
**Thyroid carcinoma**	0.088	0.182	0.105	0.245	0.200	0.521	0.291	0.558
**Pancreatic adenocarcinoma**	0.000	0.000	0.020	0.500	0.486	0.610	0.627	0.696
**Uterine Corpus Endometrial Carcinoma**	0.252	0.284	0.325	0.221	0.669	0.709	0.684	0.730
**Stomach adenocarcinoma**	0.357	0.303	0.232	0.277	0.758	0.777	0.817	0.824
**Prostate adenocarcinoma**	0.130	0.213	0.206	0.415	0.550	0.669	0.824	0.834
**Colon adenocarcinoma**	0.673	0.536	0.645	0.477	0.830	0.837	0.834	0.843
**Bladder Urothelial Carcinoma**	0.115	0.429	0.077	0.500	0.810	0.808	0.839	0.845
**Kidney renal papillary cell carcinoma**	0.574	0.684	0.618	0.627	0.875	0.881	0.841	0.848
**Lung adenocarcinoma**	0.113	0.536	0.135	0.400	0.822	0.830	0.853	0.857
**Esophageal carcinoma**	0.022	1.000	0.000	NA	0.795	0.805	0.854	0.853
**Adrenocortical carcinoma**	0.409	0.750	0.409	0.692	0.911	0.909	0.856	0.864
**Head and Neck squamous cell carcinoma**	0.280	0.298	0.333	0.352	0.834	0.840	0.872	0.876
**Cervical squamous cell carcinoma** **and endocervical adenocarcinoma**	0.042	0.250	0.097	0.318	0.855	0.858	0.875	0.876
**Kidney Chromophobe**	0.882	0.789	0.882	0.682	0.879	0.888	0.879	0.889
**Breast invasive carcinoma**	0.665	0.365	0.482	0.519	0.862	0.865	0.881	0.883
**Liver hepatocellular carcinoma**	0.150	0.750	0.390	0.513	0.844	0.851	0.884	0.884
**Rectum adenocarcinoma**	0.027	0.250	0.000	0.000	0.916	0.916	0.886	0.889
**Uterine Carcinosarcoma**	0.000	NA	0.000	NA	0.893	0.886	0.893	0.885
**Skin Cutaneous Melanoma**	0.212	0.833	0.297	0.833	0.847	0.853	0.900	0.902
**Kidney renal clear cell carcinoma**	0.746	0.585	0.754	0.537	0.881	0.888	0.902	0.907
**Lung squamous cell carcinoma**	0.488	0.438	0.545	0.493	0.860	0.868	0.910	0.910
**Brain Lower Grade Glioma**	0.478	0.637	0.463	0.441	0.865	0.864	0.911	0.913
**Pheochromocytoma and Paraganglioma**	0.435	0.833	0.391	0.857	0.922	0.923	0.911	0.914
**Glioblastoma multiforme**	0.783	0.675	0.811	0.577	0.929	0.930	0.944	0.944
**Ovarian serous cystadenocarcinoma**	0.321	0.863	0.372	0.879	0.937	0.937	0.957	0.956
**Normal**	1.000	0.836	0.999	0.832	0.994	0.993	0.983	0.982

The performance of the KNN model and the optimal performance of the random forest model—based on the point on the ROC curve nearest to 100% specificity and sensitivity—per cancer type are listed at the 100 Mb and 5 Mb segment size thresholds. PPV: Positive predictive value; TPR: True positive rate.

Finally, we utilized a random forest classification model to simulate the (near) optimal classification performance of ctDNA detectable CNVs [28]. The random forest was also readily capable of detecting the presence of cancer as expected: TPR of 0.854 and PPV of 0.997 at 100 Mb ctDNA CNV resolution and TPR of 0.895 and PPV of 0.995 at 5 Mb resolution (Fig 4B). The genomic regions deemed important by the random forest for cancer classification are presented in S2 Table.

**Fig 5:**
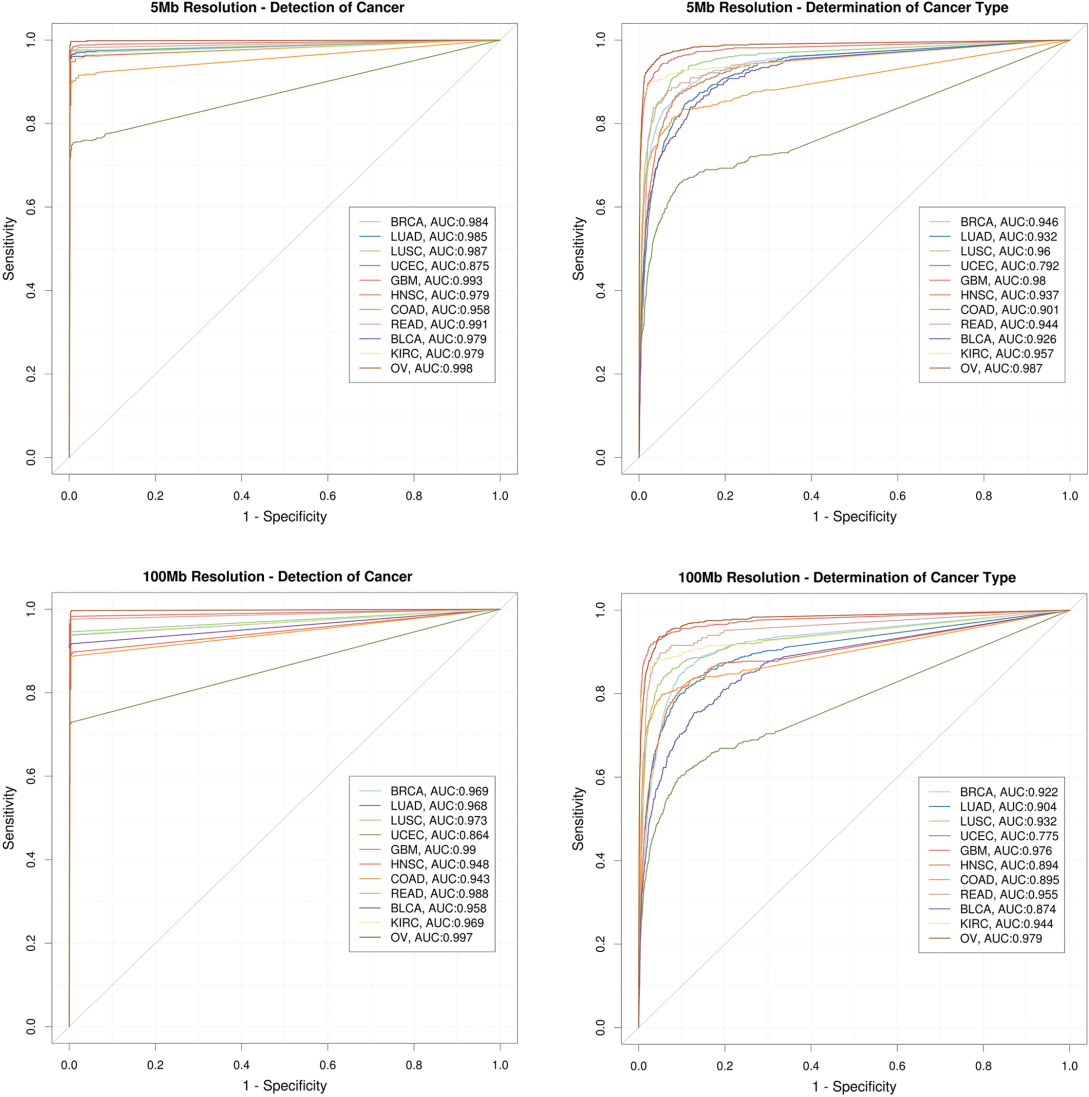
Tumor classification performance. ROC curves at 5Mb (top panels) and 100Mb ctDNA CNV resolution (bottom panels) showing performance of cancer detection (left panels) and cancer type determination (right panels) for 11 major types of solid tumors—breast adenocarcinoma (BRCA), lung adenocarcinoma (LUAD), lung squamous cell carcinoma (LUSC), uterine corpus endometrial carcinoma (UCEC), glioblastoma multiforme (GBM), head and neck squamous cell carcinoma (HNSC), colon and rectal carcinoma (COAD, READ), bladder urothelial carcinoma (BLCA), kidney renal clear cell carcinoma (KIRC), ovarian serous carcinoma (OV). A small overall increase in the AUC values when going from a 100 Mb resolution to 5 Mb resolution can be observed for both detection of cancer and determination of cancer type.

When the random forest was utilized to determine cancer type, the model had an overall accuracy of 0.78 ± 0.0054 and 0.74 ± 0.0095 at ctDNA CNV resolution of 5Mb and 100Mb respectively (Fig 4B). While there is an overall improvement in the performance of the model across most cancer types when ctDNA CNV resolution is improved from 100 Mb to 5 Mb, once again the improvement is not dramatic. ROC curves at 5Mb and 100Mb resolution are plotted in Fig 5 for the 11 major solid tumor types, and demonstrate significant differences in performance across tumor types. Certain cancer types, such as OV, BRCA, GBM and KIRC are consistently and accurately (~95%) assigned to the correct tumor type regardless of ctDNA CNV resolution. While others, apparently those of squamous histology, such as HNSC, LUSC, and BLCA show a considerable improvement (~5% increase in accuracy) in classification accuracy as ctDNA CNV detection resolution is improved. Thus, while cancer profiles can be readily distinguished from normal profiles, determination of the tissue of origin shows variability in performance across tumor types and ctDNA CNV resolution.

The optimal performance for the different cancer types, based on the point on the ROC curve nearest to 100% specificity and sensitivity is presented in Table 1 and S3 Table. Most cancer types demonstrate a PPV of >80% at even coarse grain resolution (100Mb) outpacing the PPV of diagnostic tests currently used in clinical practice. OV and GBM demonstrate the best performance (>90% TPR and PPV) suggesting they are excellent candidates for ctDNA CNV based screening applications. Many other tumor types, especially BRCA, UCEC, READ, KIRC, and LUSC also demonstrate good TPR and PPV rates (Table 1).

### Tumor misclassification

**Fig 6:**
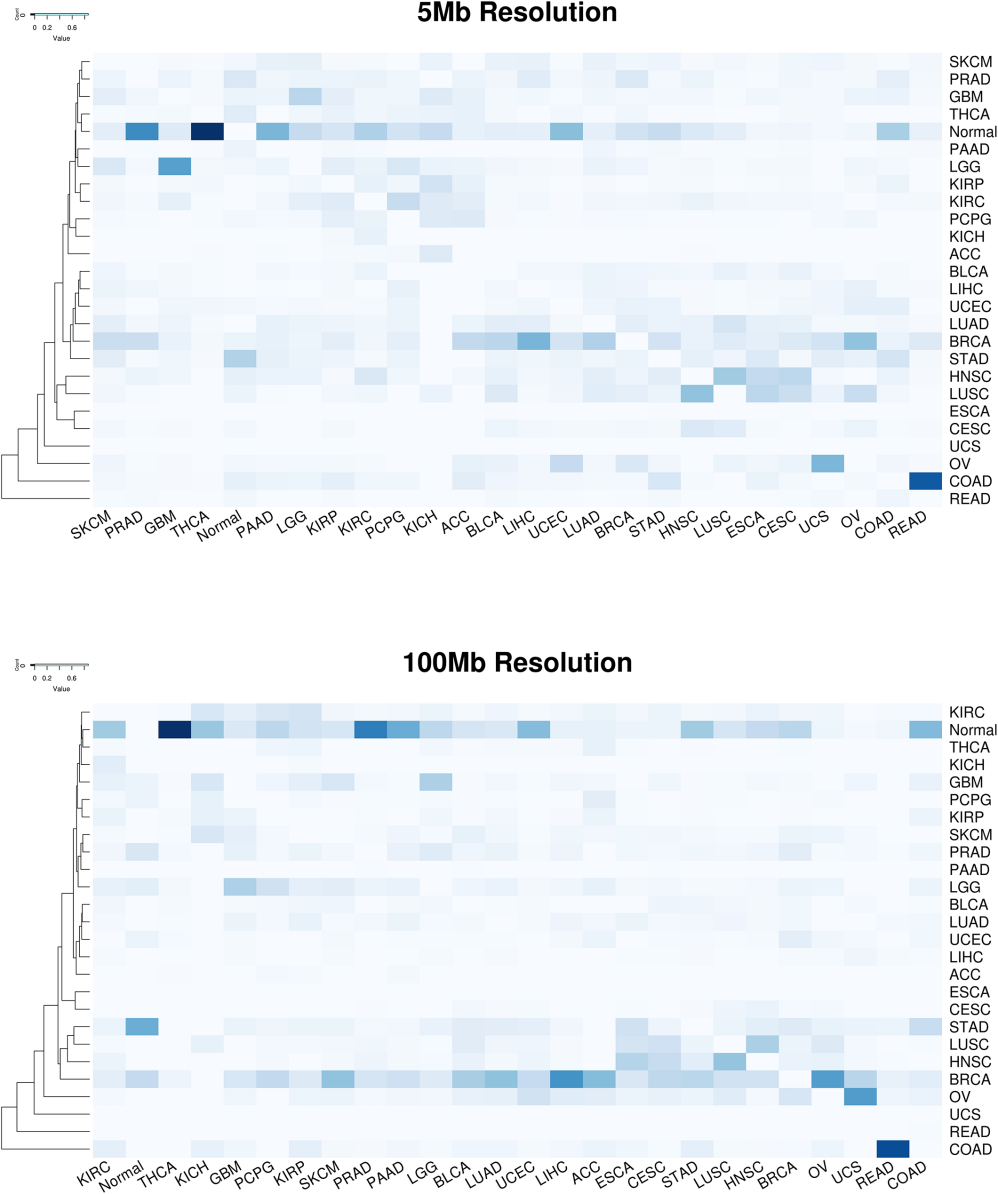
Cancer type misclassification heatmap. The frequency of cross misclassifications is depicted in a heatmap for 5 Mb (panel A) and 100 Mb (panel B) ctDNA CNV resolution. Columns correspond to the known cancer type and rows correspond to the predicted cancer type. Misclassification frequency is depicted by the darkness of each cell, with darker color reflected a higher misclassification frequency. Correct classifications are set to 0. White = 0% misclassification. Dark blue = 100% misclassification.

Finally, we investigated whether there were any underlying patterns to the tumor type misclassifications. The misclassification heat maps at 5Mb and 100 Mb ctDNA CNV resolution are displayed in Fig 6. Raw counts for misclassifications are presented in S4 Table. Many misclassifications were overt errors—cancer samples being classified as normal samples (23.4% of errors) or as breast invasive carcinomas (12.8% of errors). These errors are largely derived from cancer types such as thyroid carcinoma that have very poor performance overall and have flat CNV profiles that are difficult to distinguish from normal samples. Breast cancers tend to be diverse with respect to their cell type of origin and contain molecularly distinct subtypes [17], and thus may mimic CNV profiles of other tumor types. While breast cancer samples themselves were classified accurately, many errors were derived from other tumor types being classified as breast cancer. Given that breast cancer was the largest sample set overall, the imbalance of tumor samples per type is potentially driving this misclassification bias.

For cancer types without flat CNV profiles, misclassification tended to cluster based on tissue of origin or molecular subtype. For example, squamous cell cancers like LUSC, HNSC, ESCA and CESC show similar misclassification patterns and are often misclassified for one another. Tumors of the gastrointestinal tract, STAD, COAD, and READ are also often misclassified for one another. Similarly, brain cancers GBM and LGG show a high degree of cross misclassification. Cancers originating from the kidneys (KIRC, KIRP, and KICH) form a misclassification cluster with ACC and PCPG. These tissues originate from the intermediate mesoderm, which potentially explains their cross misclassifications. Finally, tumors driven by similar genetic mechanisms, e.g. OV and BRCA, were often misclassified for one another. These results suggest that misclassification is often biologically driven and follow clinically addressable patterns.

## Discussion

The promise of improved cancer outcomes via early detection has been hampered by the high false positive rates associated with modern cancer screening techniques. This is likely due to the fact that many current cancer screening techniques rely on correlative rather than causal biomarkers. On the other hand, ctDNA based cancer screening techniques have the potential to be highly accurate due to the fact that they directly interrogate the causal genomic drivers of tumorigenesis.

However, the unbiased nature of ctDNA sequencing can create challenges for clinical follow-up given that individual cancer mutations, especially point mutations, are not quite as cancer type specific as serum tumor markers. Since there is a large degree of overlap in the recurrent point mutations that drive common cancer types [14, 15], point mutations tend to be the least useful molecular determinant of tissue of origin [17]. When coupled with the challenges of mutation detection due to the low fraction of ctDNA amongst the total circulating DNA pool, point mutation detection likely has limited practical application for cancer screening. On the other hand, we have demonstrated that even coarse resolution ctDNA CNV detection can be used to, theoretically, both detect the presence of cancer and predict the tissue of origin. These ctDNA CNVs act as causal biomarkers for tumorigenesis. While the predictive segments described herein were selected via an unbiased methodology, about 80% of the 5 Mb segments deemed informative by the random forest model overlap with a recurrent pan-cancer or cancer-type specific amplification or deletion [26]. These segments contain known cancer genes including MYC, EGFR, ERBB2 CDKN2A, RB1 and STK11 (S5 Table). Thus, ctDNA CNVs are a promising biomarker for cancer screening that reflect the underlying molecular pathogenesis of cancer. It should be noted that other structural variant types could potentially add to the predictive power of this approach, but would be more difficult to detect given the short fragment size of ctDNA.

Our analyses also suggest that the sequencing depth required to achieve good biomarker performance is not unreasonable. While the cost of sequencing has not dropped to the point where such screening tests could be performed routinely and universally, it is feasible that these tests could be performed in high risk populations. Moreover, we are likely to reach the tipping point for sequencing costs that make universal screening feasible in the relatively near future.

A combination of ctDNA CNV and point mutation detection is likely the optimal solution for true universal screening. Many of the tumor types that performed poorly in this analysis, for example melanoma and pancreatic adenocarcinoma, are characterized by highly recurrent point mutations (BRAF V600E for melanoma and KRAS activating mutations for pancreatic adenocarcinoma) that could be detected via high sensitivity targeted assays. Thus, a combination of ctDNA CNV and targeted point mutation detection has the potential to be a very powerful cancer screening methodology. However, it is interesting to note that the tumor types that could be most effectively identified via their ctDNA CNV profile were not necessarily C-class (copy number variant driven) tumor types (as opposed to M-class (point mutation driven)) as described in another pan-cancer study [29]. For example, although KIRC is an M-class tumor, it was among the most effectively classified tumor types based on its CNV profile. While KIRC is not broadly copy number aberrant, loss of the short arm of chromosome 3 (3p) [30] containing genes like VHL, PBRM1, BAP1 and SETD2, is highly recurrent (90% of KIRC cases) and specific to KIRC. Thus, the promise of this approach is not necessarily limited to C-class tumor types.

Some potential challenges for the implementation of ctDNA CNV detection for early cancer screening are not fully addressed by this *in silico* analysis. For example, it will be necessary to understand and override the issue of sample variability in order to achieve the accurate identification of CNVs via ctDNA in practice. Moreover, it is presumed, but not known, whether CNVs predictive of cancer are actually present in early stage tumors. The battery of tumor profiles used in this analysis, derived from The Cancer Genome Atlas (http://cancergenome.nih.gov/), contain many late stage tumors. Thus, the true power of this technique will not be known until prospective clinical trials are executed. The analyses presented herein stand as a proof of concept that further studies have promise and should be attempted in diverse and larger patient cohorts.

## Methods

### Theoretical power of ctDNA CNV detection

We collected blood from 9 healthy donors in Cell-Free DNA BCT Streck tubes. Study participants were enrolled and informed consent obtained under study IRB-15-6661 approved by the Scripps Institutional Review Board. Plasma from blood samples was isolated by centrifugation at 820g for 10 minutes, then a subsequent 10-minute centrifugation at 16000g to further reduce cellular contamination. 8–10 ng/mL of cfDNA was isolated from the plasma using Qiagen’s Circulating Nucleic acid kit. cfDNA libraries were prepared and sequenced across 2 rapid mode flowcells of a HiSeq2500 to generate ~100 million 100bp paired end reads per sample. Reads were processed to remove adapter contamination using Trimmomatic [31], duplicates reads were removed using Picard (http://broadinstitute.github.io/picard/) and finally, reads were aligned to the human genome build hg38 using the BWA-mem algorithm [32]. The genome was divided into adjacent 10 Kb bins and calculated read counts per bin were determined using HTSeq-Count [33].

To account for and remove genomic regions prone to mismapping and other biases, the distribution of read counts for well-behaved genomic bins was determined using a robust Minimum Covariance Determinant estimator to determine a robust location and scale estimate of the expected read counts per bin [34]. Briefly, the MCD method looks for the h (>n/2) observations (out of n) whose classical covariance matrix has the lowest possible determinant. The estimate of location is then the average of these h points, whereas the estimate of scatter is their covariance matrix, multiplied by a consistency factor. To identify outlier bins, we calculated the Mahalanobis distance for each genomic bin based on the raw estimate of the location and scatter and removed bins with a distance greater than 15 (S2B Fig). Out of the 275,645 total bins 38,323 were marked as outliers using this approach.

Finally, to correct for GC bias, we modified the method proposed by Fan et. Al. [35] by fitting a LOESS curve to estimate the relationship between read count and GC content of the genomic bins. This relationship was utilized to normalize read counts for GC bias. The negative binomial and Poisson distribution was fit to these filtered and normalized read counts per bin.

To calculate the theoretical limit of detecting CNVs we used the following representation of the negative binomial probability mass function (pmf):

$$P(X=k)={\left(\frac{r}{r+m}\right)}^{r}\frac{\Gamma (r+k)}{k!\Gamma (r)}{\left(\frac{m}{r+m}\right)}^{k}$$

Where *r* is referred to as the “dispersion parameter” or the “shape parameter” and *m* is the mean of the distribution. The variance for this model is given by $\left(m+\frac{m^{2}}{r}\right)$. To determine the variance, we empirically determined the values of *m* and *r* at different read depths by subsampling the healthy donor cfDNA data. We then calculated the expected number of reads that would align to a bin assuming it was either affected by one copy or 4 copy change. This was done for ctDNA percentages representing a range of ctDNA burden observed in real samples. The p-value for detection of an affected bin was calculated as the right tail of the negative binomial pmf at the expected read count value calculated above. The p-value threshold for detection was set at 0.01.

### Cancer ctDNA CNV simulation

We downloaded whole genome copy number variation data, generated by the Tumor Cancer Genome Atlas Research Network (http://cancergenome.nih.gov/), for 25 different cancer types: Adrenocortical carcinoma (ACC), Bladder Urothelial Carcinoma (BLCA), Brain Lower Grade Glioma (LGG), Breast invasive carcinoma (BRCA), Cervical squamous cell carcinoma and endocervical adenocarcinoma (CESC), Colon adenocarcinoma (COAD), Esophageal carcinoma (ESCA), Glioblastoma multiforme (GBM), Head and Neck squamous cell carcinoma (HNSC), Kidney Chromophobe (KICH), Kidney renal clear cell carcinoma (KIRC), Kidney renal papillary cell carcinoma (KIRP), Liver hepatocellular carcinoma (LIHC), Lung adenocarcinoma (LUAD), Lung squamous cell carcinoma (LUSC), Ovarian serous cystadenocarcinoma (OV), Pancreatic adenocarcinoma (PAAD), Pheochromocytoma and Paraganglioma (PCPG), Prostate adenocarcinoma (PRDA), Rectum adenocarcinoma (READ), Skin Cutaneous Melanoma (SKCM), Stomach adenocarcinoma (STAD), Thyroid carcinoma (THCA), Uterine Carcinosarcoma (UCS) and Uterine Corpus Endometrial Carcinoma (UCEC). The data was accessed in December 2014 from *http*:*//gdac*.*broadinstitute*.*org/* (doi:10.7908/C19P30S6).

Specifically, we downloaded the segmentation files which contain information about the copy number of segmented genomic data produced by various algorithms like GLAD and CBS [36, 37]. CNVs in each sample were run through the SG-ADVISER CNV annotation pipeline [38] and variants with an allele frequency of >1% in the 1000 Genomes [39] or the Wellderly [40, 41] cohorts were filtered out to remove germline variants.

To model whether tumor-derived CNVs would be detectable within cfDNA at the 5 and 100 Mb resolution thresholds described in *Results*, we divided the human genome into the 5 and 100 Mb segments and for each of the segment calculated the average segment duplication value. If the average segment duplication value exceeded 1 extra copy–that segment was considered detectable. For example, if a 5 Mb segment has 5 extra copies of a 1Mb subsection or 1 extra copy spanning the entire segment then the 5 Mb segment would be considered detectable. This continuous numerical representation was then transformed using a Symbolic Aggregate Approximation [42] to a discrete representation by mapping the average segment duplication values to categorical values a cardinality of 5. In other words, the average segment duplication value was simplified to represent segments with near normal copy number, 1 copy amplification, 2 or more copy amplification, 1 copy deletion, or 2 copy deletion.

### Prediction methods

The transformed ctDNA CNV data was used to determine whether tumor profiles could be differentiated from normal sample profiles and whether tumor profiles could be distinguished from one another.

Simple clustering was done using a modified hamming distance metric where the distance between samples was calculated as the sum of the deviation between their copy number values at corresponding genomic segments. Segments with adjacent copy number values (i.e. normal and 1 extra copy, 1 extra copy and 2 extra copies, etc.) were assigned a distance value of 0.5 and larger divergence in copy number was set to a distance of 1. Hierarchical clustering was performed in R. Clustering was performed using the distance calculated for only the genomic segments deemed as important for differentiation by the random forest model described below.

The standard k-Nearest Neighbor algorithm was performed using custom R code. The modified hamming distance values described above where utilized to determined distances between samples. For each test sample, the *k* nearest other samples, where *k* was set as the square root of the total number of samples, were utilized to vote for the classification of the test sample. The test sample was assigned the majority class among the *k* nearest neighbor samples. All genomic segments were utilized for this prediction.

The discrete genomic segment values were utilized as predictors in a random forest model with 10 fold cross-validation. The random forest consisted of 100 trees, and the optimal number of variables randomly sampled as candidates at each split in the trees was determined heuristically. The ‘caret’ library in R was used to train the random forest models.

For the tumor misclassification heat maps, samples were clustered according to a custom similarity metric S. Let A and B be two tumor samples with A_n_ and B_n_ being the number of samples of tumor type A and B respectively. If α and β are the fraction of samples of A classified as B and the fraction of samples of B classified as A respectively, and N is the total number of samples, the similarity S(A,B) between A and B can be defined as -

$$S(A,B)=\frac{\left(\alpha +\beta \right)}{\frac{\left(A_{n}+B_{n}\right)}{N}}$$

### Software

Data filtration and symbolic aggregate approximation transformations were performed using custom scripts in python. All models and R calculations were performed using R v3.1.1. ROC curves denoting the performance of the models were plotted using the library ‘pROC’. Heat maps were plotted using the ‘gplots’ library.

## Supporting information

S1 Fig Scatter plot of the number of reads (log10) aligned to each 10Kb bin vs the genomic location. Each chromosome is plotted in a separate panel.
(TIFF)

S2 Fig a) Histogram of the Mahalanobis distance of each 10Kb bin from the central location estimate. b) Scatter plot of the number of reads (log10) aligned to each 10 Kb bin vs the genomic location after outliers were removed. Each chromosome is plotted in a separate panel.
(TIF)

S3 Fig Scatter plot of read counts vs the GC percentage of each 10Kb bin. Blue line shows the results of fitting a LOESS curve to the data.
(TIFF)

S4 Fig ROC curves for the random forest model at a) 5Mb resolution depicting the cancer detection for each cancer type b) 100Mb resolution depicting the cancer detection for each cancer type c) 5Mb resolution depicting the cancer type prediction for each cancer type d) 100Mb resolution depicting the cancer type prediction for each cancer type.
(TIF)

S1 Table Performance of detecting cancer using KNN. (XLSX)

S2 Table Overall accuracy values for predicting cancer type using KNN. (XLSX)

S3 Table Optimal performance calculated as the elbow point on the ROC curves for random forest models. (XLSX)

S4 Table Cancer classification and misclassification at 5 Mb segment size. (XLSX)

S5 Table Importance of bins in classification. (XLSX)

## References

[1] Suh KS, Park SW, Castro A, Patel H, Blake P, Liang M, et al Ovarian cancer biomarkers for molecular biosensors and translational medicine. Expert review of molecular diagnostics. 2010;10(8):1069–83. doi: 10.1586/erm.10.87 . 21080822 10.1586/erm.10.87

[2] Menon U, Gentry-Maharaj A, Hallett R, Ryan A, Burnell M, Sharma A, et al Sensitivity and specificity of multimodal and ultrasound screening for ovarian cancer, and stage distribution of detected cancers: results of the prevalence screen of the UK Collaborative Trial of Ovarian Cancer Screening (UKCTOCS). The Lancet Oncology. 2009;10(4):327–40. doi: 10.1016/S1470-2045(09)70026-9 . 19282241 10.1016/S1470-2045(09)70026-9

[3] Hubbard RA, Kerlikowske K, Flowers CI, Yankaskas BC, Zhu W, Miglioretti DL. Cumulative probability of false-positive recall or biopsy recommendation after 10 years of screening mammography: a cohort study. Annals of internal medicine. 2011;155(8):481–92. doi: 10.7326/0003-4819-155-8-201110180-00004 ; PubMed Central PMCID: PMC3209800. 22007042 10.1059/0003-4819-155-8-201110180-00004 PMC3209800

[4] Imperiale TF, Ransohoff DF, Itzkowitz SH, Levin TR, Lavin P, Lidgard GP, et al Multitarget stool DNA testing for colorectal-cancer screening. The New England journal of medicine. 2014;370(14):1287–97. doi: 10.1056/NEJMoa1311194 . 24645800 10.1056/NEJMoa1311194

[5] Punglia RS, D'Amico AV, Catalona WJ, Roehl KA, Kuntz KM. Effect of verification bias on screening for prostate cancer by measurement of prostate-specific antigen. The New England journal of medicine. 2003;349(4):335–42. doi: 10.1056/NEJMoa021659 . 12878740 10.1056/NEJMoa021659

[6] Buys SS, Partridge E, Black A, Johnson CC, Lamerato L, Isaacs C, et al Effect of screening on ovarian cancer mortality: the Prostate, Lung, Colorectal and Ovarian (PLCO) Cancer Screening Randomized Controlled Trial. JAMA. 2011;305(22):2295–303. doi: 10.1001/jama.2011.766 . 21642681 10.1001/jama.2011.766

[7] Newman AM, Bratman SV, To J, Wynne JF, Eclov NC, Modlin LA, et al An ultrasensitive method for quantitating circulating tumor DNA with broad patient coverage. Nature medicine. 2014;20(5):548–54. doi: 10.1038/nm.3519 ; PubMed Central PMCID: PMCPMC4016134. 24705333 10.1038/nm.3519 PMC4016134

[8] Dawson SJ, Tsui DW, Murtaza M, Biggs H, Rueda OM, Chin SF, et al Analysis of circulating tumor DNA to monitor metastatic breast cancer. The New England journal of medicine. 2013;368(13):1199–209. doi: 10.1056/NEJMoa1213261 . 23484797 10.1056/NEJMoa1213261

[9] Bettegowda C, Sausen M, Leary RJ, Kinde I, Wang Y, Agrawal N, et al Detection of circulating tumor DNA in early- and late-stage human malignancies. Science translational medicine. 2014;6(224):224ra24 doi: 10.1126/scitranslmed.3007094 ; PubMed Central PMCID: PMCPMC4017867. 24553385 10.1126/scitranslmed.3007094 PMC4017867

[10] Crowley E, Di Nicolantonio F, Loupakis F, Bardelli A. Liquid biopsy: monitoring cancer-genetics in the blood. Nat Rev Clin Oncol. 2013;10(8):472–84. doi: 10.1038/nrclinonc.2013.110 . 23836314 10.1038/nrclinonc.2013.110

[11] Heidary M, Auer M, Ulz P, Heitzer E, Petru E, Gasch C, et al The dynamic range of circulating tumor DNA in metastatic breast cancer. Breast Cancer Res. 2014;16(4):421 doi: 10.1186/s13058-014-0421-y ; PubMed Central PMCID: PMCPMC4303230. 25107527 10.1186/s13058-014-0421-y PMC4303230

[12] Chan KC, Jiang P, Zheng YW, Liao GJ, Sun H, Wong J, et al Cancer genome scanning in plasma: detection of tumor-associated copy number aberrations, single-nucleotide variants, and tumoral heterogeneity by massively parallel sequencing. Clin Chem. 2013;59(1):211–24. doi: 10.1373/clinchem.2012.196014 . 23065472 10.1373/clinchem.2012.196014

[13] Leary RJ, Sausen M, Kinde I, Papadopoulos N, Carpten JD, Craig D, et al Detection of chromosomal alterations in the circulation of cancer patients with whole-genome sequencing. Science translational medicine. 2012;4(162):162ra54 doi: 10.1126/scitranslmed.3004742 ; PubMed Central PMCID: PMC3641759. 23197571 10.1126/scitranslmed.3004742 PMC3641759

[14] Shlien A, Malkin D. Copy number variations and cancer. Genome medicine. 2009;1(6):62 doi: 10.1186/gm62 ; PubMed Central PMCID: PMC2703871. 19566914 10.1186/gm62 PMC2703871

[15] Kandoth C, McLellan MD, Vandin F, Ye K, Niu B, Lu C, et al Mutational landscape and significance across 12 major cancer types. Nature. 2013;502(7471):333–9. doi: 10.1038/nature12634 ; PubMed Central PMCID: PMC3927368. 24132290 10.1038/nature12634 PMC3927368

[16] Carrasco DR, Tonon G, Huang Y, Zhang Y, Sinha R, Feng B, et al High-resolution genomic profiles define distinct clinico-pathogenetic subgroups of multiple myeloma patients. Cancer Cell. 2006;9(4):313–25. doi: 10.1016/j.ccr.2006.03.019 . 16616336 10.1016/j.ccr.2006.03.019

[17] Hoadley KA, Yau C, Wolf DM, Cherniack AD, Tamborero D, Ng S, et al Multiplatform analysis of 12 cancer types reveals molecular classification within and across tissues of origin. Cell. 2014;158(4):929–44. doi: 10.1016/j.cell.2014.06.049 ; PubMed Central PMCID: PMC4152462. 25109877 10.1016/j.cell.2014.06.049 PMC4152462

[18] Hofree M, Shen JP, Carter H, Gross A, Ideker T. Network-based stratification of tumor mutations. Nature methods. 2013;10(11):1108–15. doi: 10.1038/nmeth.2651 ; PubMed Central PMCID: PMCPMC3866081. 24037242 10.1038/nmeth.2651 PMC3866081

[19] Heitzer E, Ulz P, Belic J, Gutschi S, Quehenberger F, Fischereder K, et al Tumor-associated copy number changes in the circulation of patients with prostate cancer identified through whole-genome sequencing. Genome medicine. 2013;5(4):30 doi: 10.1186/gm434 ; PubMed Central PMCID: PMCPMC3707016. 23561577 10.1186/gm434 PMC3707016

[20] Kirkizlar E, Zimmermann B, Constantin T, Swenerton R, Hoang B, Wayham N, et al Detection of Clonal and Subclonal Copy-Number Variants in Cell-Free DNA from Patients with Breast Cancer Using a Massively Multiplexed PCR Methodology. Transl Oncol. 2015;8(5):407–16. doi: 10.1016/j.tranon.2015.08.004 ; PubMed Central PMCID: PMCPMC4631096. 26500031 10.1016/j.tranon.2015.08.004 PMC4631096

[21] Bianchi DW, Chudova D, Sehnert AJ, Bhatt S, Murray K, Prosen TL, et al Noninvasive Prenatal Testing and Incidental Detection of Occult Maternal Malignancies. JAMA. 2015;314(2):162–9. doi: 10.1001/jama.2015.7120 . 26168314 10.1001/jama.2015.7120

[22] Amant F, Verheecke M, Wlodarska I, Dehaspe L, Brady P, Brison N, et al Presymptomatic Identification of Cancers in Pregnant Women During Noninvasive Prenatal Testing. JAMA Oncol. 2015;1(6):814–9. doi: 10.1001/jamaoncol.2015.1883 . 26355862 10.1001/jamaoncol.2015.1883

[23] Lefkowitz RB, Tynan JA, Liu T, Wu Y, Mazloom AR, Almasri E, et al Clinical validation of a noninvasive prenatal test for genomewide detection of fetal copy number variants. Am J Obstet Gynecol. 2016 doi: 10.1016/j.ajog.2016.02.030 . 26899906 10.1016/j.ajog.2016.02.030

[24] Nygren AOH, Dean J, Jensen TJ, Kruse S, Kwong W, van den Boom D, et al Quantification of Fetal DNA by Use of Methylation-Based DNA Discrimination. Clinical Chemistry. 2010;56(10):1627–35. doi: 10.1373/clinchem.2010.146290 20729299 10.1373/clinchem.2010.146290

[25] Fan HC, Gu W, Wang J, Blumenfeld YJ, El-Sayed YY, Quake SR. Non-invasive prenatal measurement of the fetal genome. Nature. 2012;487(7407):320–4. doi: 10.1038/nature11251 ; PubMed Central PMCID: PMC3561905. 22763444 10.1038/nature11251 PMC3561905

[26] Zack TI, Schumacher SE, Carter SL, Cherniack AD, Saksena G, Tabak B, et al Pan-cancer patterns of somatic copy number alteration. Nature genetics. 2013;45(10):1134–40. doi: 10.1038/ng.2760 ; PubMed Central PMCID: PMC3966983. 24071852 10.1038/ng.2760 PMC3966983

[27] Beroukhim R, Mermel CH, Porter D, Wei G, Raychaudhuri S, Donovan J, et al The landscape of somatic copy-number alteration across human cancers. Nature. 2010;463(7283):899–905. doi: 10.1038/nature08822 ; PubMed Central PMCID: PMCPMC2826709. 20164920 10.1038/nature08822 PMC2826709

[28] Fernández-Delgado M, Cernadas E, Barro S, Amorim D. Do we Need Hundreds of Classifiers to Solve Real World Classification Problems?. Journal of Machine Learning Research. 2014;15:3133–81.

[29] Ciriello G, Miller ML, Aksoy BA, Senbabaoglu Y, Schultz N, Sander C. Emerging landscape of oncogenic signatures across human cancers. Nature genetics. 2013;45(10):1127–33. doi: 10.1038/ng.2762 ; PubMed Central PMCID: PMC4320046. 24071851 10.1038/ng.2762 PMC4320046

[30] Zbar B, Brauch H, Talmadge C, Linehan M. Loss of alleles of loci on the short arm of chromosome 3 in renal cell carcinoma. Nature. 1987;327(6124):721–4. doi: 10.1038/327721a0 . 2885753 10.1038/327721a0

[31] Bolger AM, Lohse M, Usadel B. Trimmomatic: a flexible trimmer for Illumina sequence data. Bioinformatics. 2014;30(15):2114–20. doi: 10.1093/bioinformatics/btu170 ; PubMed Central PMCID: PMCPMC4103590. 24695404 10.1093/bioinformatics/btu170 PMC4103590

[32] Li H, Durbin R. Fast and accurate short read alignment with Burrows-Wheeler transform. Bioinformatics. 2009;25(14):1754–60. doi: 10.1093/bioinformatics/btp324 ; PubMed Central PMCID: PMCPMC2705234. 19451168 10.1093/bioinformatics/btp324 PMC2705234

[33] Anders S, Pyl PT, Huber W. HTSeq—a Python framework to work with high-throughput sequencing data. Bioinformatics. 2015;31(2):166–9. doi: 10.1093/bioinformatics/btu638 ; PubMed Central PMCID: PMCPMC4287950. 25260700 10.1093/bioinformatics/btu638 PMC4287950

[34] Rousseeuw PJ, Hubert M. Robust statistics for outlier detection. Wiley Interdisciplinary Reviews: Data Mining and Knowledge Discovery. 2011;1(1):73–9. doi: 10.1002/widm.2

[35] Fan HC, Quake SR. Sensitivity of noninvasive prenatal detection of fetal aneuploidy from maternal plasma using shotgun sequencing is limited only by counting statistics. PloS one. 2010;5(5):e10439 doi: 10.1371/journal.pone.0010439 ; PubMed Central PMCID: PMCPMC2862719. 20454671 10.1371/journal.pone.0010439 PMC2862719

[36] Hupe P, Stransky N, Thiery JP, Radvanyi F, Barillot E. Analysis of array CGH data: from signal ratio to gain and loss of DNA regions. Bioinformatics. 2004;20(18):3413–22. doi: 10.1093/bioinformatics/bth418 . 15381628 10.1093/bioinformatics/bth418

[37] Olshen AB, Venkatraman ES, Lucito R, Wigler M. Circular binary segmentation for the analysis of array-based DNA copy number data. Biostatistics. 2004;5(4):557–72. doi: 10.1093/biostatistics/kxh008 . 15475419 10.1093/biostatistics/kxh008

[38] Erikson GA, Deshpande N, Kesavan BG, Torkamani A. SG-ADVISER CNV: copy-number variant annotation and interpretation. Genetics in medicine: official journal of the American College of Medical Genetics. 2014 doi: 10.1038/gim.2014.180 . 25521334 10.1038/gim.2014.180 PMC4886732

[39] Genomes Project C, Abecasis GR, Auton A, Brooks LD, DePristo MA, Durbin RM, et al An integrated map of genetic variation from 1,092 human genomes. Nature. 2012;491(7422):56–65. doi: 10.1038/nature11632 ; PubMed Central PMCID: PMC3498066. 23128226 10.1038/nature11632 PMC3498066

[40] Borrell B. Sequencing projects bring age-old wisdom to genomics. Nature medicine. 2011;17(11):1329 doi: 10.1038/nm1111-1329a . 22064397 10.1038/nm1111-1329a

[41] Singleton MV, Guthery SL, Voelkerding KV, Chen K, Kennedy B, Margraf RL, et al Phevor combines multiple biomedical ontologies for accurate identification of disease-causing alleles in single individuals and small nuclear families. American journal of human genetics. 2014;94(4):599–610. doi: 10.1016/j.ajhg.2014.03.010 ; PubMed Central PMCID: PMC3980410. 24702956 10.1016/j.ajhg.2014.03.010 PMC3980410

[42] Lin J, Keogh E, Lonardi S, Chiu B. A Symbolic Representation of Time Series, with Implications for Streaming Algorithms. In proceedings of the 8th ACM SIGMOD Workshop on Research Issues in Data Mining and Knowledge Discovery. 2003.

