# Supplementary figures and images for: Assessment of circulating copy number variant detection for cancer screening
Source: PLoS One. 2017 Jul 7;12(7):e0180647. doi: 10.1371/journal.pone.0180647 (PMC5501586; doi:10.1371/journal.pone.0180647)

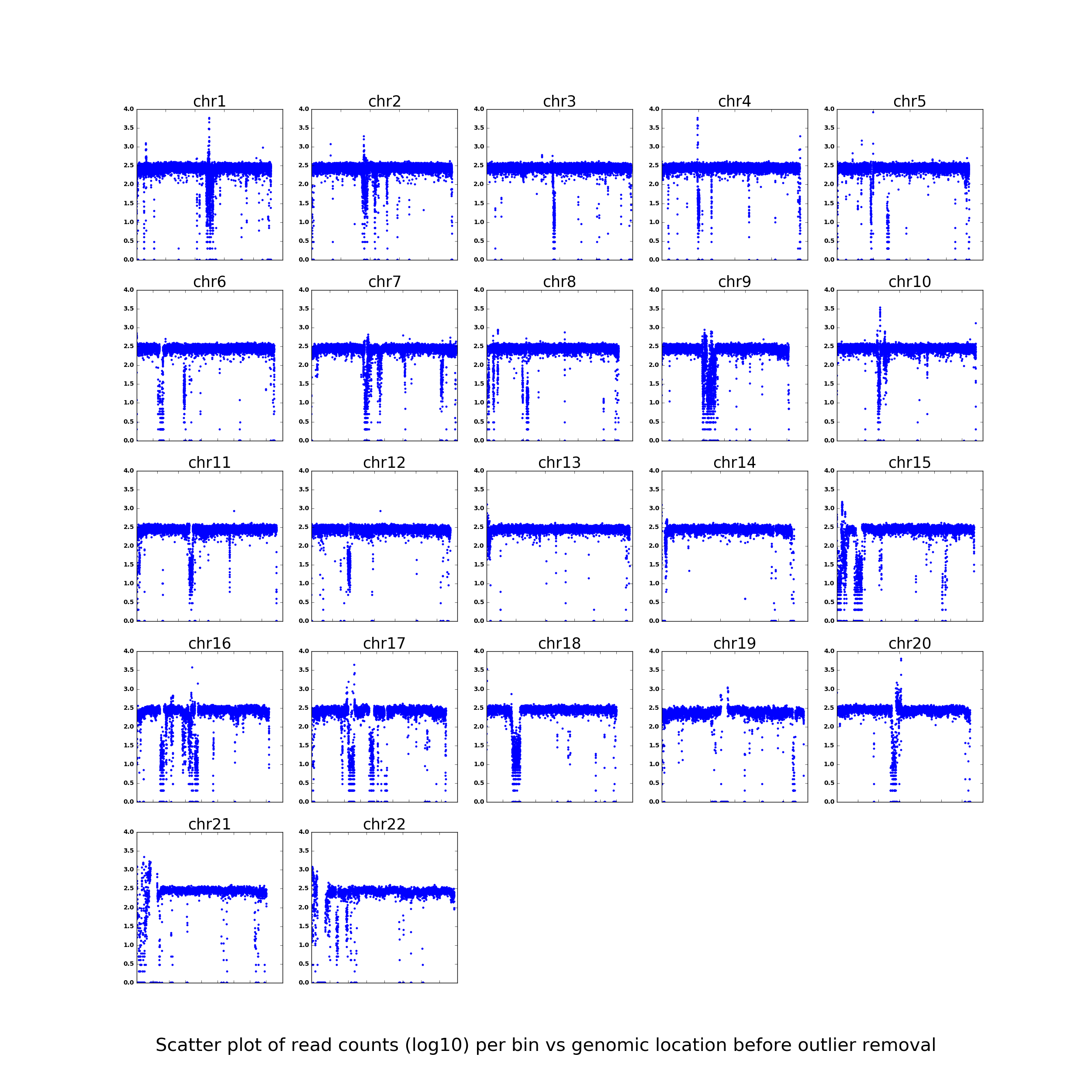

Supplement: S1 Fig — Each chromosome is plotted in a separate panel. (TIFF) [file pone.0180647.s001.tiff]

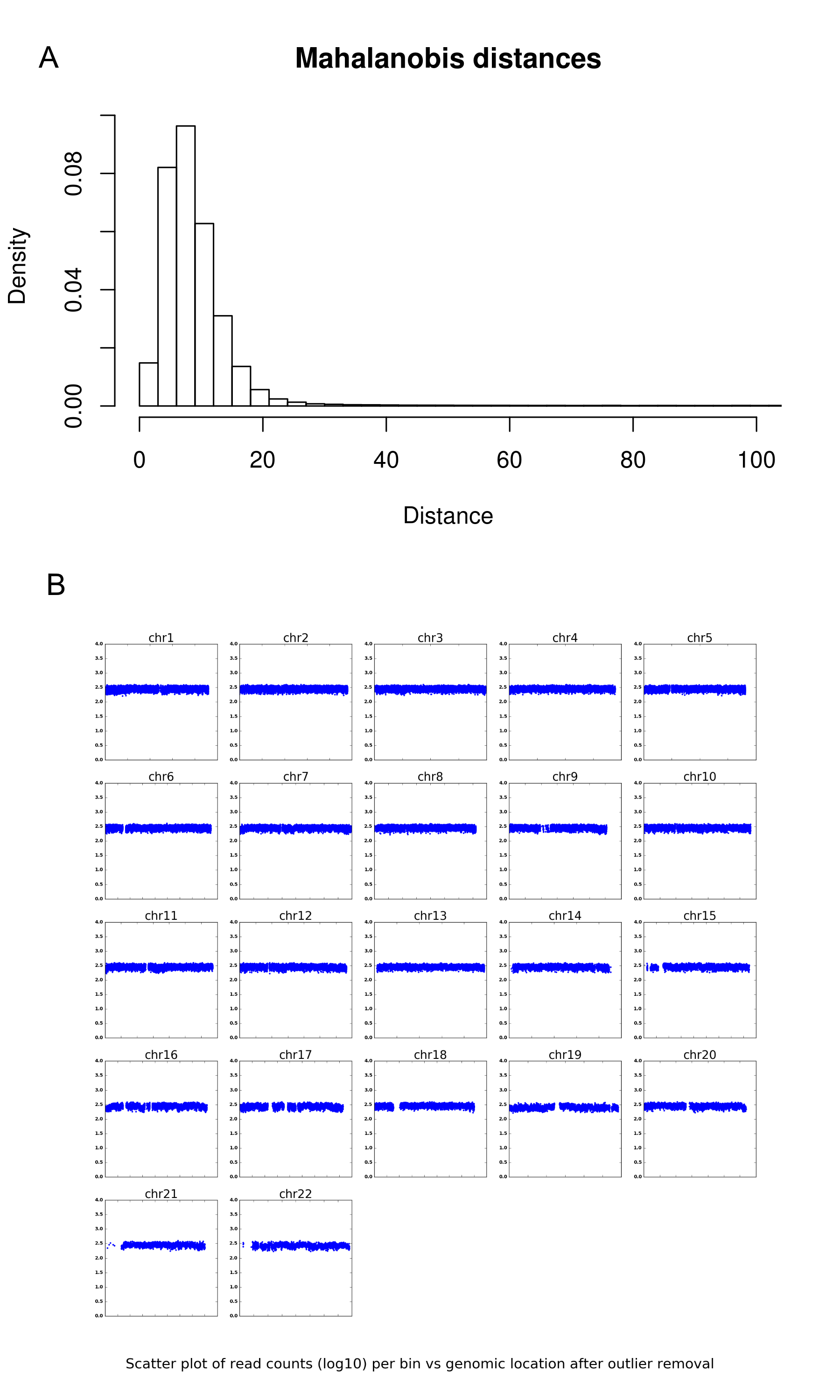

Supplement: S2 Fig — a) Histogram of the Mahalanobis distance of each 10Kb bin from the central location estimate. b) Scatter plot of the number of reads (log10) aligned to each 10 Kb bin vs the genomic location after outliers were removed. Each chromosome is plotted in a separate panel. (TIF) [file pone.0180647.s002.tif]

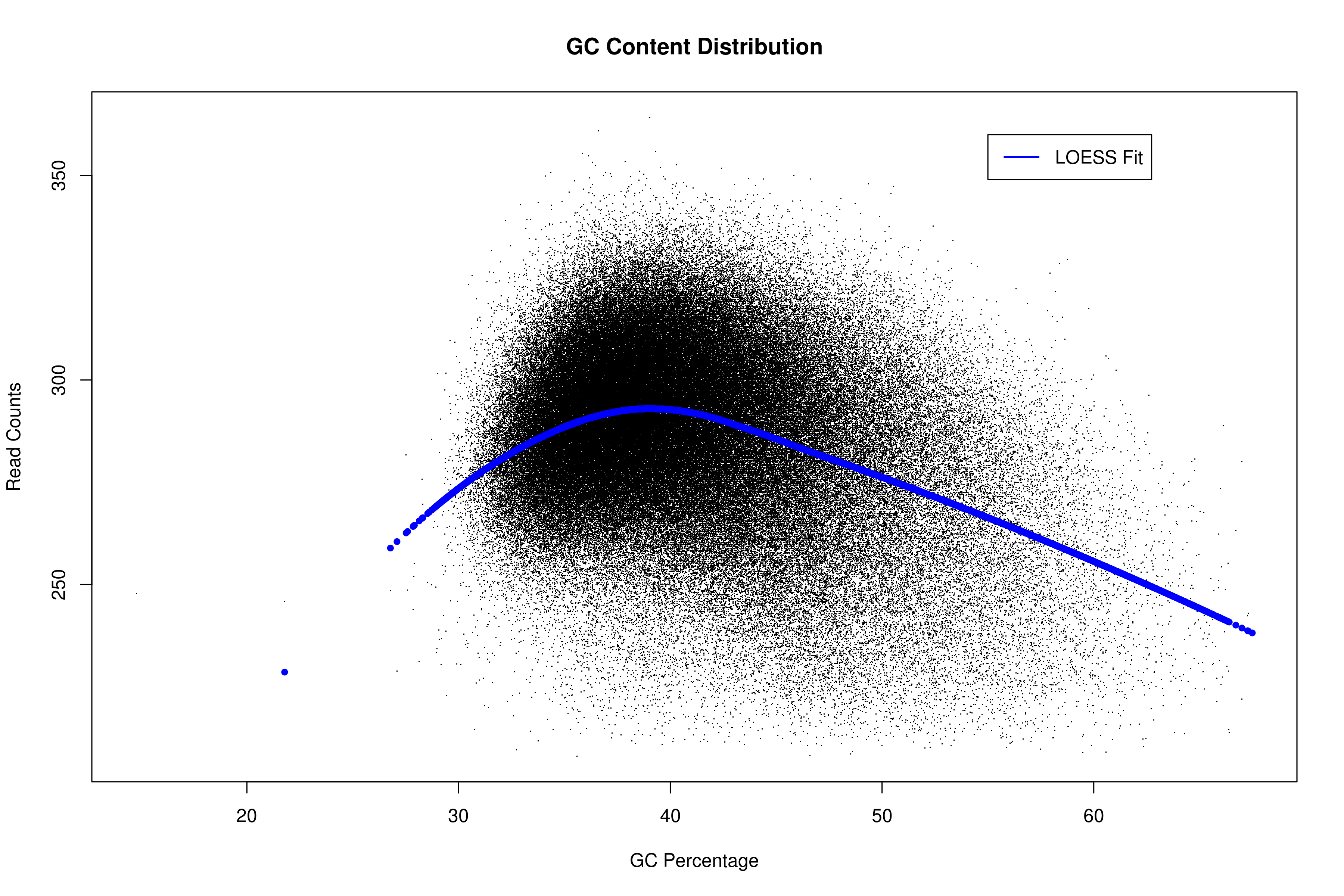

Supplement: S3 Fig — Blue line shows the results of fitting a LOESS curve to the data. (TIFF) [file pone.0180647.s003.tiff]
